# Construction of Silver–Calcium Micro-Galvanic Cell on Titanium for Immunoregulation Osteogenesis

**DOI:** 10.34133/bmef.0173

**Published:** 2025-09-08

**Authors:** Zhenhao Hou, Xingdan Liu, Xianming Zhang, Ji Tan, Xuanyong Liu

**Affiliations:** ^1^ State Key Laboratory of High Performance Ceramics, Shanghai Institute of Ceramics, Chinese Academy of Sciences, Shanghai 200050, China.; ^2^ Center of Materials Science and Optoelectronics Engineering, University of Chinese Academy of Sciences, Beijing 100049, China.; ^3^ School of Chemistry and Materials Science, Hangzhou Institute for Advanced Study, University of Chinese Academy of Sciences, Hangzhou 310024, China.

## Abstract

**Objective:** This work aims to construct a functional titanium surface with spontaneous electrical stimulation for immune osteogenesis and antibacteria. **Impact Statement:** A silver–calcium micro-galvanic cell was engineered on the titanium implant surface to spontaneously generate microcurrents for osteoimmunomodulation and bacteria killing, which provides a promising strategy for the design of a multifunctional electroactive titanium implant. **Introduction:** Titanium-based implants are usually bioinert, which often leads to inflammation-induced loosening. Electrical stimulation has therapeutic potential; however, its dependence on external devices limits its clinical application. Therefore, designing an electroactive titanium surface with endogenous electrical stimulation capability is a promising strategy to overcome implant failure induced by inflammation. **Methods:** The silver–calcium micro-galvanic cell was constructed on titanium substrate surfaces by the ion implantation technique. RAW264.7 and MC3T3-E1 were used for cell culture studies with the material to evaluate immunomodulatory and osteogenic abilities of the implant. The expression levels of inflammatory genes and voltage-gated Ca^2+^ channel-related genes were tested for investigating the mechanism of immunoregulation. The antibacterial properties of the modified titanium were assessed. Finally, its immunomodulatory effects in vivo were verified by a mouse subcutaneous inflammation model. **Results:** The silver–calcium micro-galvanic modified titanium surface generates microcurrents and releases Ca^2+^, which induces macrophage polarization toward the M2 phenotype and promotes osteogenic differentiation via paracrine signaling, exhibiting excellent antibacterial activity. **Conclusion:** The silver–calcium micro-galvanic cell on titanium could regulate the immune response to promote bone repair and exhibit antibacterial capabilities through noninvasive electrical stimulation, providing a promising strategy for the design of multifunctional electroactive implant surfaces.

## Introduction

Dental implant technology is now emerging as the centerpiece of oral rehabilitation treatment, with an explosion of technology and applications [1,2]. Titanium, a common metal implant, is widely used in dentistry because of its good biocompatibility and stable chemical properties [3]. However, titanium-based metal implants currently in clinical use are biologically inert without antibacterial and osteogenic properties [4,5]. They may still fail after implantation surgery due to bacterial infection and poor integration [6–10]. Most past studies have focused on the direct action of materials on osteoblasts or bacteria, [11,12] and have neglected the influence of materials on the immune response in vivo, which is crucial to the practical application effect of the implant [13]. In some conditions, over-immunization of the organism can even produce inflammation-induced progressive bone destruction [14]. Osteogenesis is a complex process, during which various biochemical reactions occur that are controlled by the body’s immune regulation, electrical stimulation (ES), and other factors [15,16]. Therefore, constructing titanium surfaces that can simultaneously achieve antibacteria, immunomodulation, and osteogenesis remains challenging.

In recent years, an increasing number of studies have focused on the modulation of immunity through physical means such as light or ultrasound and chemical means such as drugs or biological reagents to enhance the osteointegration of materials. Researchers have induced the conversion of macrophages to M2 type by the thermal effect produced by excitation of the modified coating with near-infrared light and ultrasonic stimulation of the material’s nanofiber hydrogel to produce a temporal release of substances as well as the release of biologically active ions and biologically active substances through the material [15,17–20]. ES as an attractive mean can directly affect the behavior of cells and has great potential in therapeutic and regenerative medicine [21]. Gu et al. [22] covered indium tin oxide planar microelectrodes with poly(vinylidene fluoride-trifluoroethylene) films and found that the material could activate the M2 polarization signaling cascade via ES-triggered ion channels. Wu et al. [23] prepared BaTiO_3_/Ti_6_Al_4_V piezoelectric scaffolds and found that the resulting piezoelectric stimuli inhibited mitogen-activated protein kinase (MAPK)/c-Jun N-terminal kinase (JNK) signaling and activated oxidative phosphorylation (OXPHOS) in macrophages, thus promoting macrophage M2 polarization. This remodeling of the immune microenvironment would greatly contribute to the osteogenic effect [24].

In addition to its effects on immunity, electrical stimulation (ES) can directly promote osteogenic differentiation of cells under appropriate conditions [22,25]. Zhang et al. [26] simulated endogenous biopotentials by incorporating barium dioxide nanoparticles in poly(vinylidene fluoridetrifluoroethylene) and found that it could promote osteogenic differentiation of bone marrow mesenchymal stem cells (BMSCs). Controllable surface potential activates transmembrane calcium channels and permits the inward flow of extracellular Ca^2+^, affecting the bioactivity and cell behavior of BMSC cells [27,28]. The combination of generating ES with the bioactive ingredient antioxidant polydopamine-modified hydroxyapatite has resulted in the preparation of periosteal materials with excellent bone-enhancing properties and immunomodulatory abilities [29]. Not only cells but also ES can markedly modulate bacterial behavior and confer excellent antibacterial ability to the materials. Wang et al. [30] prepared a novel capacitive antibacterial dressing by combining polypyrrole-wrapped carbon cloth and bacterial cellulose hydrogel, and found that the material exhibited high bactericidal efficiency under 1-V ES for 10 min. Ren et al. [31] prepared a piezoelectric dual-network nanofiber dressing using polyvinylidene difluoride (PVDF), which was shown to have good antibacterial effects against *Staphylococcus aureus*. Herewith, constructing the coating with ES on titanium implants has a great strategy for immune regulation, osteogenesis, and antibacterial effects.

Traditionally, common ES is direct coupling with an external electric field, but this type of coupling has many limitations for organism applications [21]. Because clinical bioelectrical stimulation involves invasive surgical implantation of metal electrodes, these electrodes can lead to serious complications and greatly reduce the quality of patient survival [32]. Therefore, it is very important to make the materials to generate ES while avoiding trauma caused by external power sources. There have been a number of studies that have mimicked the biological internal electric field by piezoelectric materials such as barium titanate to generate ES to influence cell behavior [33,34]. However, piezoelectric materials need to rely on deformation to convert mechanical energy into electrical energy. This process requires active or passive force on the wound site, which can adversely affect tissue healing and growth. Therefore, it is important to make the material generate ES spontaneously to avoid the trauma caused by external power supply.

There are many disadvantages of external power stimulation, so designing a surface with intrinsic ES is a better choice. In recent years, a variety of metal micro-galvanic cell structures have been designed, and Spisak et al. [35,36] investigated copper–zinc 2-electrode and copper–bismuth–zinc 3-electrode systems, and found that they have good antibacterial ability. This construction of a micro-galvanic cell on the surface of the material using differences in the order of metal activity allows the material to generate a spontaneous electric current to electrically stimulate the cells. In this process, the material surface also releases the corresponding metal ions. Metal elements are essential components of life and are involved in almost all biochemical reactions in living organisms. Ions, as the main form of metal present in living organisms, have a notable impact on a variety of cells. By studying the effects of metal ions on cells, it has been found that they play an important role in bone regeneration and immune induction [37–39]. Among all types of metallic elements, calcium is the most abundant element in the human body [37]. Many studies have shown that calcium ions can induce gene expression or signal to promote osteogenic differentiation of BMSCs extracellularly [40,41]. Gavilán et al. [42] found that calcium ions promote cell migration and adhesion during the early stage of implantation, and then regulate the behavior of osteoblasts through the phosphatidylinositol 3-kinase (PI3K)–Akt signal pathway in the later stage of implantation. On the other hand, Ca^2+^ plays an important role in the immune system. Zhang et al. [43] found that Ca^2+^ promotes M2 macrophage polarization by activating calcium-sensitive receptor (CaSR)-activated Arg1 and interleukin-10 (IL-10) transcription in macrophages. Ca^2+^ may also activate the PI3K–Akt and adenosine 3′,5′-monophosphate (cAMP)–protein kinase A (PKA) pathways to promote M2 polarization and facilitate bone regeneration by building a suitable immune microenvironment [44].

Therefore, in this study, silver–calcium (Ag–Ca) micro-galvanic cells were constructed on titanium surface by plasma immersion ion implantation (PIII) technique. Spontaneous microcurrents were caused to be generated on the surface of titanium material to study the immunizing effect of ES generated by silver and calcium particles on the titanium surface. Macrophages (RAW264.7), mouse calvaria-derived pre-osteoblastic cell subclone E1 (MC3T3-E1), and mouse BMSCs (mBMSCs) were used as experimental subjects to investigate the effect of this material system on immunity and the alteration of the osteogenic effect by culturing the macrophages alone as well as coculturing the macrophages with other cells. In addition, the material was implanted subcutaneously in mice to explore the immune effects of the material in the organism. This study may provide new insights into the immunological studies of multi-element doping of metal surfaces.

## Results

### Surface characterization

**Fig. 1.**
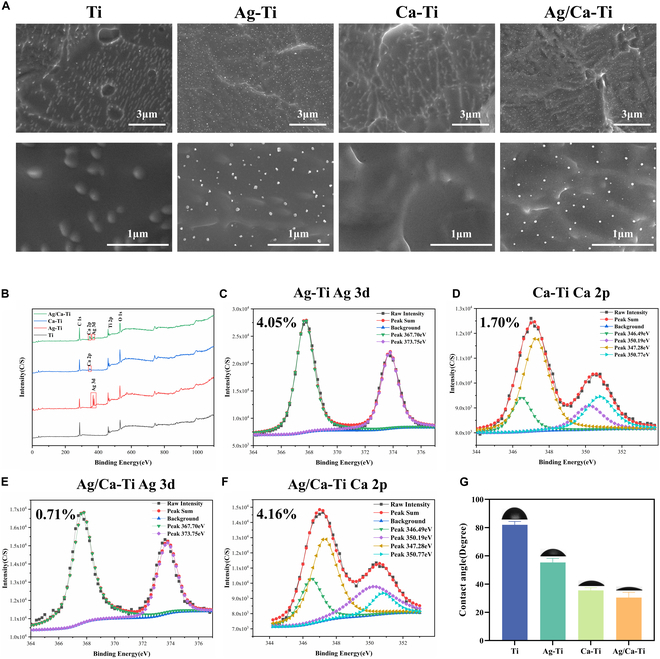
Characterization of the surface properties of the samples. (A) SEM images of surface morphology at low and high magnification. (B) XPS full spectrum of Ti, Ag–Ti, Ca–Ti, and Ag/Ca–Ti samples. (C) XPS fine spectrum of Ag 3d in Ag–Ti group. (D) XPS fine spectrum of Ca 2p in Ca–Ti group. XPS fine spectrum of (E) Ag 3d and (F) Ca 2p in Ag/Ca–Ti. (G) Surface contact angle of the various samples.

Figure 1A shows the photographs of the surface microstructure of the Ag/Ca-PIII system at 10,000 and 50,000 times, where the Ti group is the sample group bombarded by Ar under the same conditions. The surface of Ti is rough with some irregular bulges. Compared with the Ti group, the Ag–Ti group had nanoscale fused particles on the surface, confirming the successful injection of silver into the surface of the Ti sheet by PIII. No obvious nanoparticles were observed on the surface of the Ca–Ti group after calcium injection. However, at high magnification, blurred boundaries of the bulge structure on the Ca–Ti surface could be observed. Ca particles could not be observed due to the low solubility of metallic calcium in titanium, which resulted in the mixing of calcium atoms with the substrate [13]. Figure 1G shows the contact angle test results of the samples, and it can be seen that the contact angle of Ca–Ti is much smaller than that of the Ti samples, which can also laterally confirm that Ca has been successfully injected into the sample surface. The size of the contact angle is Ti > Ag–Ti > Ca–Ti > Ag/Ca–Ti, which might be caused by the activation of the titanium surface through ion implantation. The Ag/Ca–Ti group can clearly see that the silver–calcium co-injection has made the Ag nanoparticles on the surface of the titanium sheet smaller and the distribution of the particles more uniform than the injection of elemental silver alone.

In order to reveal the chemical state and chemical composition of each sample surface of the Ag/Ca–PIII system, the results of the x-ray photoelectron spectroscopy (XPS) spectra analysis of each sample are shown in Fig. 1B to F. As shown in Fig. 1B, only the peaks of Ti 2p and O 1s were detected in the Ti sample. From the figures, it can be seen that the Ag/Ca–Ti sample surface was successfully loaded with 2 elements, Ag and Ca. In Ag–Ti and Ag/Ca–Ti samples, in addition to the peaks of Ti 2p and O 1s, the peaks of Ag 3d were detected, corresponding to metallic silver and its oxides (Fig. 1C and E). In addition, dual peaks of Ca 2d, corresponding to calcium and its oxides, were detected on the Ca–Ti and Ag/Ca–Ti surfaces (Fig. 1D and F), which also indicates successful loading of calcium onto the sample surface [45].

**Fig. 2.**
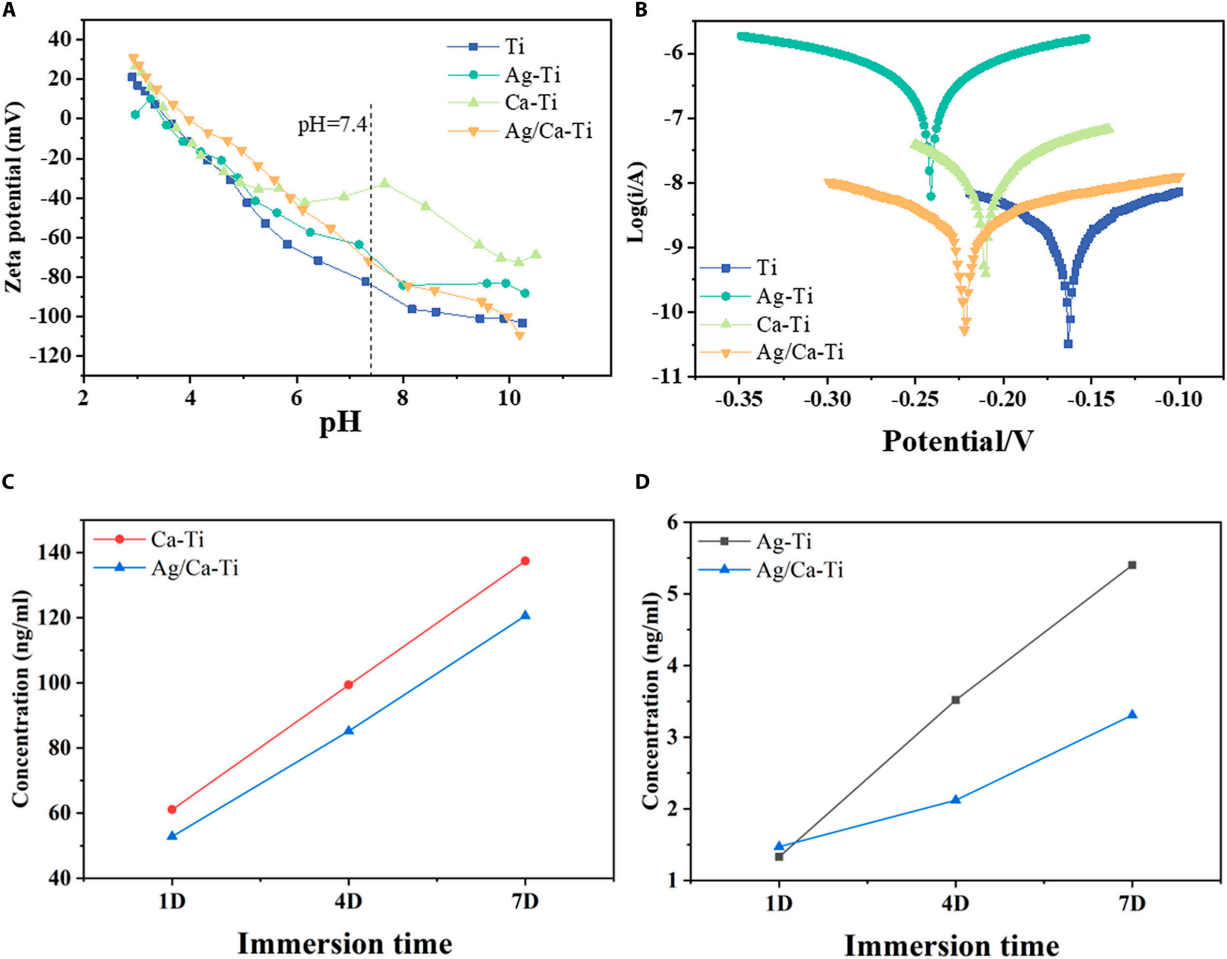
Physical and chemical properties of samples. (A) Surface zeta potential of various samples. (B) Surface kinetic potential polarization curves of various samples. (C) Calcium ions and (D) silver ion release of each group in saline solution after 1, 4, and 7 d of immersion.

Figure 2A presents the zeta potential of the samples across a pH range of 3.0 to 10.0. As the pH increases, the zeta potential of the sample surface decreases. The pH value in the human physiological environment is about 7.4, at which time the zeta potentials of all 4 groups of samples are negative. The zeta potential values of the samples after ion implantation increased compared to the Ti group. The dynamic potential polarization tests can indicate the corrosion resistance as well as the chemical reaction activity of the samples, which were tested in saline at 0.9 wt % NaCl, and the results are shown in Fig. 2B. The trend of corrosion potential for each sample is Ti > Ca–Ti > Ag/Ca–Ti > Ag–Ti, and the trend of corrosion current is Ag–Ti > Ca–Ti > Ag/Ca–Ti > Ti. Due to the injection of Ag and Ca on the surface of Ti, it is more prone to electrochemical reactions compared to the dense TiO_2_ film. Since Ca injection will form a layer of calcium oxide on the surface [46], a small amount of Ca(OH)_2_ may be generated to cover the surface of the samples after the reaction, which results in slightly higher corrosion resistance of Ag/Ca–Ti compared with Ag–Ti. This is also corroborated with the ion release results of the samples. The release profiles of calcium ions from Ca–Ti and Ag/Ca–Ti samples were comparable (Fig. 2C). In contrast, the release of silver ions from Ag–Ti and Ag/Ca–Ti samples exhibited a time-dependent divergence (Fig. 2D), consistent with the observed difference in corrosion potential, where Ag–Ti displayed a lower corrosion potential than Ag/Ca–Ti.

### Biocompatibility evaluation

**Fig. 3.**
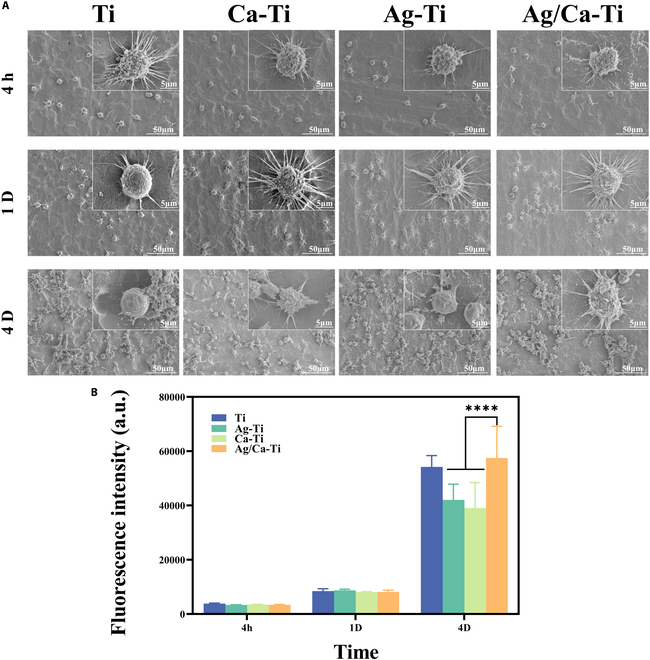
(A) Morphology and (B) proliferation of macrophages cultured on the sample surfaces for 4 h, 1 d, and 4 d (***P < 0.001, ****P < 0.0001).

In order to evaluate the cytotoxicity of the samples and their biocompatibility, the mouse-derived mononuclear macrophage leukemia cells (RAW264.7) were cultured on the surface of the materials. After dehydration and drying, the scanning electron microscopy (SEM) morphology of the cells on the sample surface was observed. Figure 3A shows the SEM images of macrophage cells. After 4 h of culture alone, some macrophages had already adhered to the sample surface, and the morphology of the cells on the surface of different samples was similar. The cells on the surface of the samples were in the shape of a polygonal star, but the pseudopods of the cells on the surface of the experimental group were short, and those of the cells on the surface of the Ti samples were long; the number of cells on the surface of the samples was obviously increased after 1 d of culture, and the cells got different degrees of spreading, and the pseudopods of different morphology were stretched out. The 3 groups of samples in the experimental group had more and longer surface cell pseudopods, and the Ti group had fewer surface cell pseudopods and a small spreading area. After 4 d of incubation, the cells were spread over the entire sample surface, with multilayered growth, and the cells formed a biofilm on the surface of the sample. The surface morphology of the macrophages on the sample surface changed noticeably. The surface cells of the samples of the various groups were spherical, and the surface cells of the rest of the samples were almost free of cellular pseudopods, except for the presence of pseudopods in the Ag/Ca–Ti group. There were almost no cell pseudopods on the surface of the samples. The proliferation results of macrophages cultured alone on the sample surface are shown in Fig. 3B. Macrophages on different sample surfaces all grew with the extension of incubation time, indicating good biocompatibility between the samples and RAW264.7. There was no significant difference in cell proliferation between the groups after 4 h and 1 d of incubation. After 4 d, the cell proliferation of the Ag–Ti and Ca–Ti groups was significantly lower than that of the Ti group, while there was no significant difference between the Ag/Ca-Ti group and Ti group for macrophages proliferation. These results indicated that the Ag/Ca–Ti group had good biocompatibility. To further confirm the cytocompatibility of the materials, the mBMSCs and the mouse calvaria-derived pre-osteoblastic cell (MC3T3-E1) were also incubated with the samples. As shown in Fig. S1, the materials exhibited no cytotoxicity toward mBMSCs and MC3T3-E1.

In conclusion, the results showed that the Ag/Ca–Ti group was biocompatible with cells. However, in macrophages, different material surfaces mediate the cells to produce different adhesion morphology, and the change of cell morphology is closely related to the polarization phenotype. Therefore, it is seen that during the sample–cell interaction, the materials mediate the polarization of macrophages into different functional phenotypes, which are involved in the bone tissue healing process.

### Immune response of macrophage

**Fig. 4.**
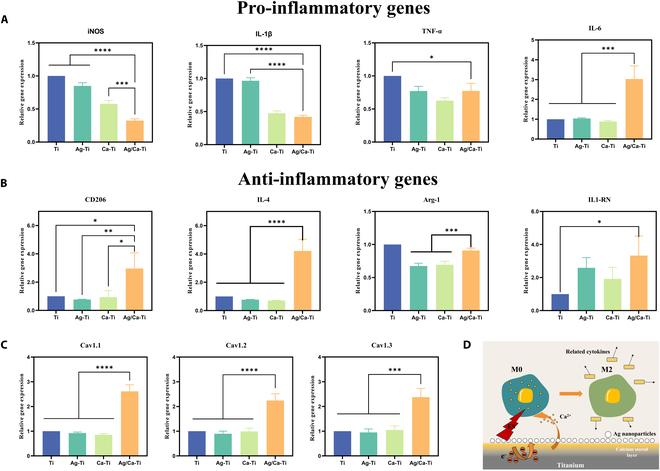
Immune response of macrophage in different groups. (A) Pro-inflammatory gene expression in macrophage after culture for 4 d. (B) Anti-inflammatory gene expression in macrophage after culture for 4 d. (C) VGCC-related gene expression in macrophage after culture for 4 d. (D) Schematic diagram of Ag–Ca micro-galvanic cell regulating the behavior of macrophages by generating microcurrent (*P < 0.05, **P < 0.01, ***P < 0.001, ****P < 0.0001).

In order to evaluate the cellular inflammatory response on the surface of each group of materials, the expression of related genes was further characterized. Figure 4A and B shows the experimental results of polymerase chain reaction (PCR) technique to detect the expression level of immune-related genes in macrophages on the surface of samples cultured for 4 d. Compared with Ti, the expression levels of inflammation-related genes in macrophages on the surface of samples from the Ag–Ti group were lower, and in general, the expression of anti-inflammatory and pro-inflammatory genes was smaller than that of Ti. The expression of inflammation-related genes in the surface cells of the samples from the Ca–Ti group was similar to that of the Ag–Ti group, and the corresponding pro-inflammatory and anti-inflammatory factors were either highly expressed or poorly expressed, showing no consistency. This indicates that the Ca–Ti group has no obvious anti-inflammatory and pro-inflammatory tendency. The expression of inflammation-related genes in the surface cells of the samples from the Ag/Ca–Ti group was low in pro-inflammatory factors and high in anti-inflammatory factors compared with the other samples, which implies a significant anti-inflammatory effect in the Ag/Ca–Ti group compared with the other samples in the group. Specifically, the associated secretion of pro-inflammatory factors showed that Ag/Ca–Ti had much lower expression of inducible nitric oxide synthase (iNOS) and tumor necrosis factor-α (TNF-α) compared to genes in the Ti group. These results indicate that the Ag/Ca–Ti group exhibits significant anti-inflammatory effects. In terms of anti-inflammatory gene expression, Ag/Ca–Ti samples also showed much higher expression of MRC1 and IL-4 than samples from other groups. When RAW264.7 was incubated on Ag/Ca–Ti samples alone, the experimental group samples showed a significant anti-inflammatory tendency.

The internal environment of living organisms is inherently complex, and immuno-osteogenesis involves the participation of multiple cell types. To further investigate how other cells influence the immune expression of macrophages, we established a coculture system of mBMSCs and RAW264.7 macrophages. The results demonstrated that macrophages maintained a strong anti-inflammatory tendency, as shown in Fig. S2.

ES activates cell surface calcium channels and regulates cell behavior by affecting calcium ion uptake [47]. Therefore, it is important to study the expression of cell surface calcium channel-related genes. Figure 4C shows the expression of voltage-gated Ca^2+^ channel (VGCC)-related genes in RAW264.7. The VGCC-related genes of Ag/Ca–Ti samples showed high expression, and the secretion levels of related cytokines were much higher than those of samples from other groups. Based on the above experimental results, it can be assumed that Ag–Ca micro-galvanic cell generating microcurrents will affect the macrophage polarization toward M2 type of inflammation suppression as shown in Fig. 4D.

### Osteogenic properties of samples

**Fig. 5.**
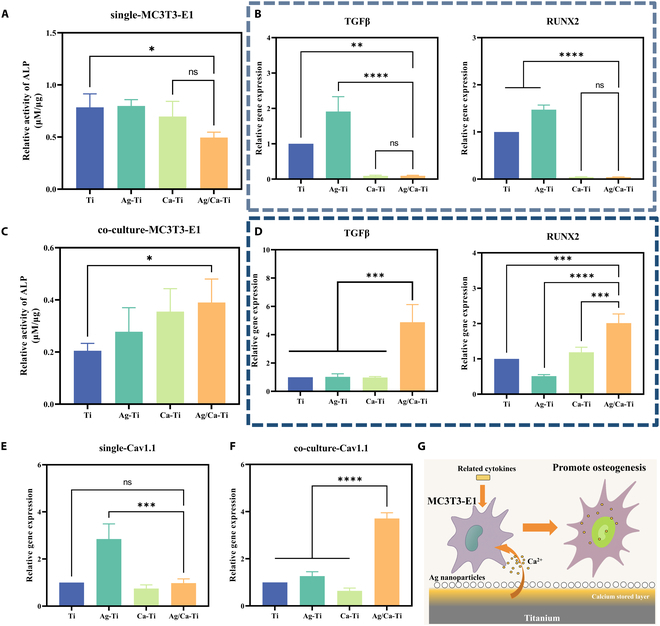
Osteogenesis of samples in vitro. (A) Relative ALP activity and (B) osteogenic gene expression in MC3T3-E1 after culture with samples for 7 d. (C) Relative ALP activity and (D) osteogenic gene expression in MC3T3-E1 after coculture with macrophage stimulated by different samples. (E) VGCC-related gene expression in MC3T3-E1 cultured on samples. (F) VGCC-related gene expression in MC3T3-E1 cocultured with macrophage on sample surfaces. (G) Schematic diagram of Ag–Ca micro-galvanic cell promoting osteogenic differentiation of MC3T3-E1 (*P < 0.05, **P < 0.01, ***P < 0.001, ****P < 0.0001).

In order to assess the promotional effect of the material on cellular osteogenesis, we chose mouse calvaria-derived pre-osteoblastic cell subclone E1 (MC3T3-E1) homologous to mouse macrophages (RAW264.7) for osteogenic assessment. MC3T3-E1 was first cultured on the surface of the samples, while RAW264.7 was added for coculturing, which was used to investigate the effect of immunization on the osteogenic effect of the cells. Figure 5A shows that when MC3T3-E1 was cultured alone, the alkaline phosphatase (ALP) content of Ag/Ca–Ti was not highly expressed but decreased compared to Ti. Figure 5B shows the expression of osteogenesis-related genes after culturing MC3T3-E1 alone. It can be seen that the expression level of osteogenic genes of MC3T3-E1 in Ag/Ca–Ti samples was significantly lower than that of MC3T3-E1 in Ti samples, and this trend was consistent with the ALP results. The above results indicated that the Ag/Ca–Ti samples did not show significant bone enhancement effect when MC3T3-E1 was cultured alone.

However, when RAW264.7 was added for coculture, a significant increase in ALP expression was found in the Ag/Ca–Ti sample group, which was significantly different from Ti (Fig. 5C). Meanwhile, the expression of MC3T3-E1 osteogenic genes was examined after coculture, as shown in Fig. 5D. The expression levels of all relevant osteogenic genes of MC3T3-E1 were up-regulated on the Ag/Ca–Ti samples, among which the expression levels of transforming growth factor β (TGFβ) and RUNX2 were several times different compared with that on the Ti samples. These results indicated that the addition of macrophages to MC3T3-E1 in Ag/Ca–Ti samples can up-regulate the expression levels of osteogenic genes in MC3T3-E1 and promote cellular osteogenesis, as shown in Fig. 5G. To explore the role of macrophages in regulating MC3T3-E1 osteogenesis, we investigated the expression of VGCC-related genes in MC3T3-E1 cultured alone and cocultured MC3T3-E1, as shown in Fig. 5E and F. The VGCC signature gene Cav1.1 showed high expression in Ag/Ca–Ti samples when coculturing MC3T3-E1, while it was not significantly elevated when cultured alone.

Different levels of ES have different effects on different cells [48]. Although the mechanism by which ES affects cells is currently unknown, it is certain that it affects cell membrane surface protein channels through changes in cell membrane potential [49].

To further determine the effect of the materials on cellular osteogenesis, mBMSCs were used in separate cultures. The results showed that the expression level of mBMSCs osteogenic genes in Ag/Ca–Ti samples was significantly higher than that in several other groups of samples, as shown in Fig. S3. MC3T3-E1 and mBMSCs exhibited different response behaviors when cultured on the surface of Ag/Ca–Ti samples, which may be related to the degree of electrical stimulation. Ag/Ca–Ti samples were produced by the formation of a micro-galvanic cell on the surface of Ti, which produces the transfer of electrons to produce a series of biological effects; however, since the degree of electron transfer is relatively fixed (with a certain current magnitude), it may produce different degrees of effects for different cells.

### Immune response in vivo

**Fig. 6.**
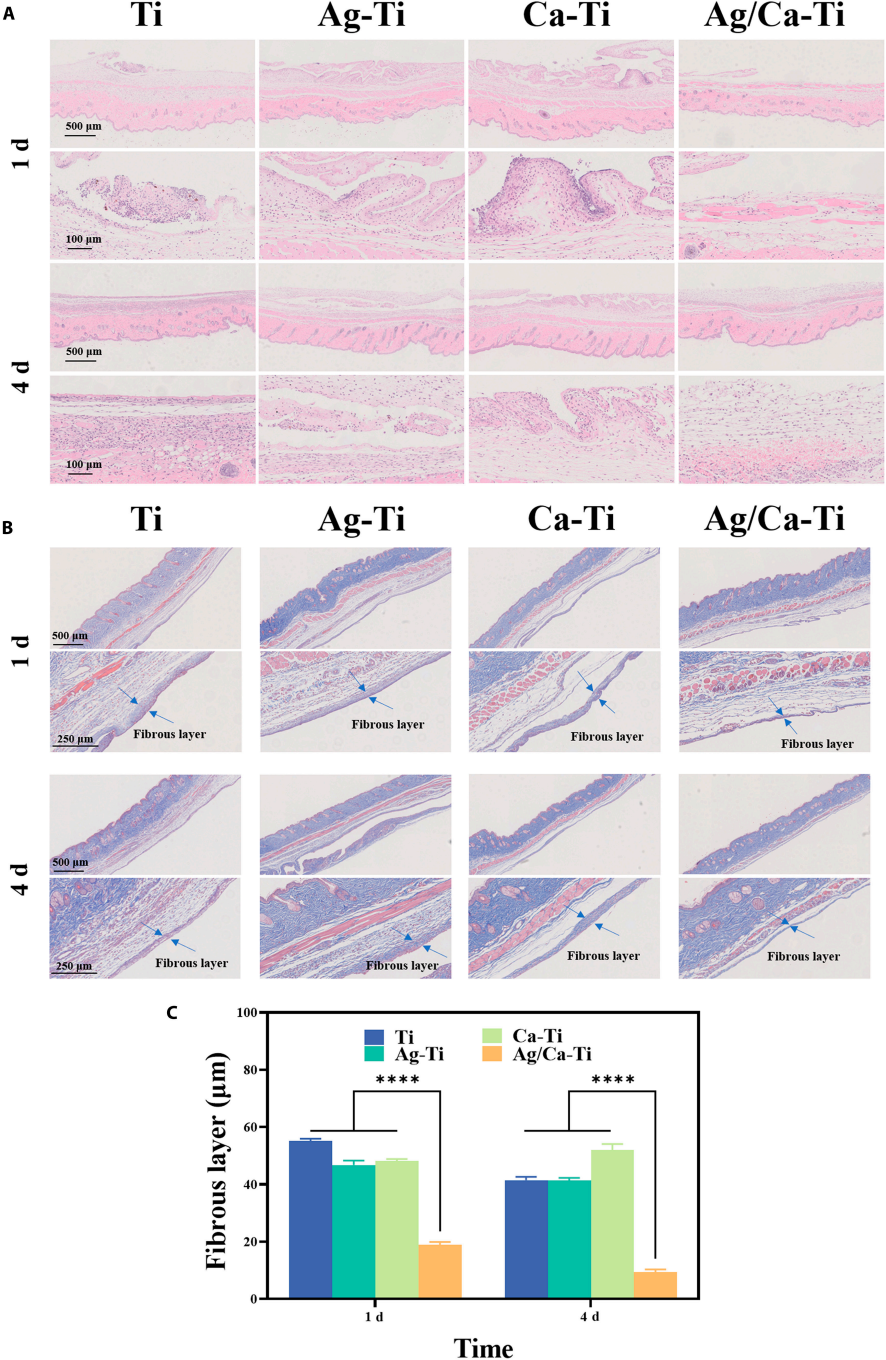
Immune response of samples in vivo. Images of (A) H&E staining and (B) Masson’s trichrome staining of air pouch tissues adjacent to different samples at 1 and 4 d after implantation and (C) the corresponding thickness of the fibrous layers on days 1 and 4.

In order to study the immunization effect of different samples in the organism, an airbag model was constructed on the back of mice to study the immune response. Figure 6A and B shows the representative images of tissues in each group with hematoxylin and eosin (H&E) and Masson trichrome staining, respectively. Figure 6C is a count of the thickness of the fiber layer. After 1 d of material implantation, a fibrous layer was formed around the samples, especially thicker for Ti and Ca–Ti, which included a large number of inflammatory cells. Compared to the other groups, Ag/Ca–Ti samples had the thinnest fibrous layer and the least distribution of inflammatory cells. After 4 d of material implantation, the fibrous layer of the samples in all groups started to become thinner and the number of inflammatory cells decreased. Compared to day 1, Ag–Ti and Ca–Ti exhibited a significant reduction in the thickness of the fibrous layer, accompanied by a substantial decline in inflammatory cell infiltration by day 4. The inflammation around Ti implant was also reduced at day 4 compared with that at day 1, but it still had a higher number of inflammatory cells. It is noteworthy that the Ag/Ca–Ti samples had the thinnest fiber layer among the group of samples in the same period, both on the first day and on the fourth day.

**Fig. 7.**
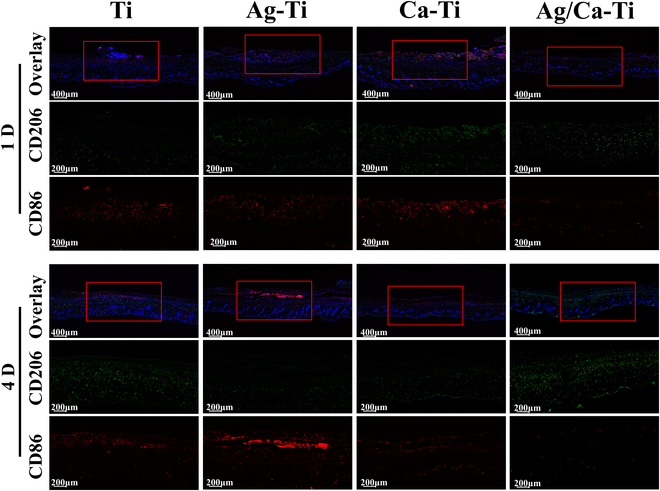
Representative images of immunofluorescence staining for labeling macrophage phenotype in tissues (M1: CD86: red, M2: CD206: green, 4′,6-diamidino-2-phenylindole: blue).

Macrophages polarize into 2 states upon stimulation, including M1 and M2 [50]. CD86 and CD206 were selected as the markers of M1 and M2 types, respectively. As shown in Fig. 7, the colabeled staining images of CD206 and CD86 showed that both of them contained a high number of inflammation-associated cells on their surfaces. The Ag/Ca–Ti samples had a lower number of inflammatory type cells, and the Ag/Ca–Ti samples showed mainly anti-inflammatory effects accompanied by a small number of pro-inflammatory-associated cells. Compared with the first day, the number of inflammation-related cells in the Ag–Ti and Ca–Ti samples on the fourth day decreased, with predominantly pro-inflammatory type of cells and a smaller number of anti-inflammatory type of cells. At the same time, the Ag/Ca–Ti samples still showed the anti-inflammatory effect of the tissue, i.e., the number of anti-inflammatory type cells was much higher than that of pro-inflammatory type cells. This result is also consistent with the result that Ag/Ca–Ti samples can reduce the inflammation of tissues in Fig. 6. Based on the results of in vivo and in vitro experiments, it can be concluded that Ag/Ca–Ti samples can effectively reduce the inflammatory response of tissues and cells, thus promoting the healing growth of tissues.

### Antibacterial effect

To further evaluate the antimicrobial properties of the materials, *S. aureus* and *Escherichia coli* were cultured on their surfaces, as illustrated in Fig. S4. The results demonstrated that both Ag–Ti and Ag/Ca–Ti samples exhibited strong antimicrobial activity. Further analysis indicated that the superior antimicrobial performance of Ag/Ca–Ti was primarily attributed to direct contact between the material surface and the bacteria. This effect may be associated with the ability of the material to influence electron transfer to the bacterial biofilm during ES.

The Ag/Ca–Ti samples were fabricated by co-implanting Ag and Ca nanoparticles into the titanium surface via PIII. The embedded Ag and Ca nanoparticles form a localized micro-galvanic cell on the Ti substrate, with the physiological environment acting as an electrolyte. This configuration enables the continuous generation of microcurrents and localized electron transfer at the material–bacteria interface.

Bacterial energy metabolism predominantly occurs at the cell membrane, where the electron transport chain (ETC) drives oxidative phosphorylation and adenosine triphosphate (ATP) production [51]. The microcurrent generated by the Ag/Ca–Ti surface induces electrochemical stress on the bacterial membrane, disrupting the natural electron flow across the ETC [52]. This interference leads to collapse of the proton motive force (PMF), impaired ATP synthesis, and, ultimately, bacterial cell death [53].

## Discussion

In this study, Ag–Ca micro-galvanic cells were constructed by injecting Ag and Ca on the Ti surface by PIII technique. This system can be simplified to be viewed as a simple primary cell structure consisting of 2 electrodes (Ag and Ca) connected by a wire (Ti) immersed in an electrolyte. Since the hydrogen standard electrode potential of Ag (+0.7996 V) is higher than that of Ca (−2.87 V), Ag serves as the cathode and Ca serves as the anode in this primary cell structure [54,55]. In a normal physiological environment (pH ≥ 7), the following reactions can occur:

Cathodic reaction:

$$2H_{2}O+2e^{-}\to H_{2}+2OH^{-}$$ (1)

$$O_{2}+2H_{2}O+4e^{-}\to 4OH^{-}$$ (2)

Anodic reaction:

$$Ca\to Ca^{2+}+2e^{-}$$ (3)

Equations 1 and 2 are electron-consuming reactions. The electrons are provided by the oxidation reaction of Ca into Ca^2+^, which is transferred to the Ag particles, and the 2 reaction processes of hydrogen precipitation and oxygen depletion take place. The OH^−^ formed by the reaction will lead to a concentration gradient layer of OH^−^ diffusion on the surface of the material. In order to maintain the current stability, every Ca^2+^ generated will produce 2 OH^−^ at the same time, so the whole electrochemical reaction process will be affected by the diffusion of Ca^2+^ and OH^−^.

Macrophages are important for promoting tissue repair and bone regeneration, and the microcurrent generated by Ag/Ca–Ti samples and the release of Ca^2+^ will inevitably affect the polarization state of macrophages. On the one hand, there are many channel proteins on the cell surface, and the permeability of the proteins can be altered under the stimulation of appropriate currents to manipulate ions in and out of the cell for the purpose of modulating cell function [56,57]. On the other hand, a strong correlation has been observed between intracellular calcium ion levels in macrophages and their functional states [58,59]. Therefore, the polarization direction of macrophages can be directly affected by influencing the activity of calcium channel proteins on the macrophage surface. For example, transient receptor potential vanilloid 1 (TRPV1) induces Ca^2+^ inward flow and inhibits macrophage polarization toward the M1 type by promoting nuclear factor-erythroid 2-related factor 2 (Nrf2) nuclear localization [60]. In addition, calcium influx into macrophages requires the large-conductance voltage and calcium-activated potassium channel (K_Ca_1.1 or BK channel), the absence of which results in the conversion of macrophages to the M1 type [61]. Therefore, Ca^2+^ uptake and inward flow can be promoted by increasing the activity of transporter proteins to inhibit the formation of M1-type macrophages. Voltage-gated Ca^2+^ channels (VGCCs) are very specialized and important ion channels that have 3 main subdivisions defined by pore-forming α_1_ subunits, Cav1.1, Cav1.2, and Cav1.3 channels [62,63].

As shown in Fig. 4C, the Ag/Ca–Ti samples exhibited the highest expression of VGCC-related genes, significantly surpassing those of Ti and other samples. This enhanced VGCC gene expression promoted the polarization of macrophages in the Ag/Ca–Ti group toward an anti-inflammatory phenotype, specifically the M2 type. This finding is consistent with the results in Fig. 4A that Ag/Ca–Ti samples can promote macrophage transformation to M2 type. Upon appropriate stimulation, the up-regulation of VGCC gene expression increases the activity of calcium channels on the macrophage membrane, leading to a specific influx of Ca^2+^ into the cell and thereby elevating intracellular calcium levels. The increased intracellular Ca^2+^ concentration activates calcium-dependent signaling pathways, such as the synthesis and secretion of prostaglandin E2 (PGE2), which has been shown to promote macrophage polarization toward the anti-inflammatory M2 phenotype [55]. The immune system, through paracrine secretion, can further influence bone formation [64]. M2 Macrophages inhibit osteoclast activity and promote osteoblast differentiation by secreting factors such as IL-10, TGF, and IL-1 [65].

**Fig. 8.**
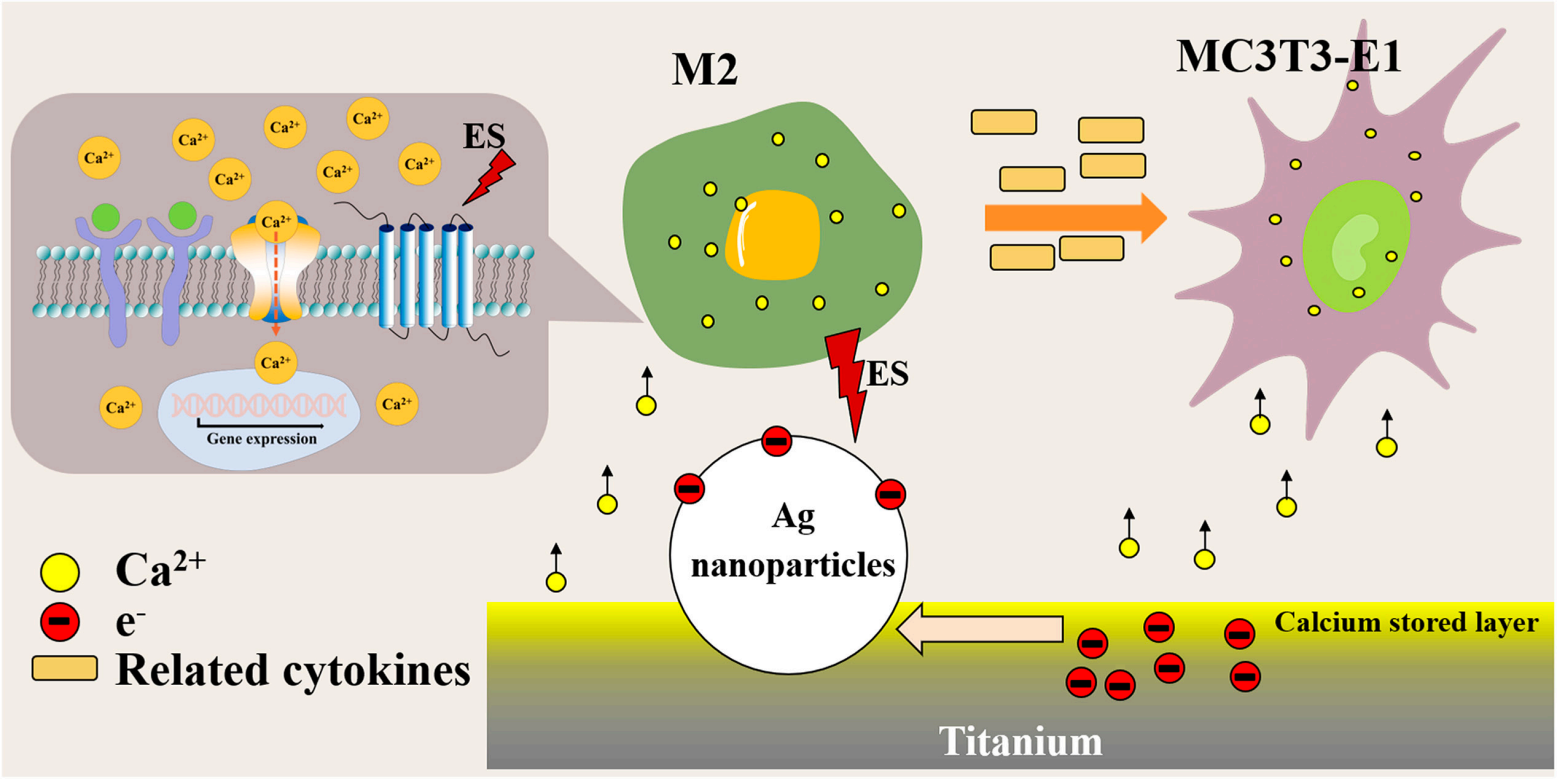
Schematic diagram of the mechanism of microcurrents on the sample surface regulating macrophages and MC3T3-E1.

For osteogenesis-associated cells, activation of VGCC channels is critical, and elevated intracellular Ca^2+^ concentrations can elicit downstream biochemical responses that promote cellular osteogenic differentiation [66,67]. As shown in Fig. 5E and F, the expression of Cav1.1 gene in MC3T3-E1 of Ag/Ca–Ti group was low when MC3T3-E1 was cultured on samples, whereas the expression of Cav1.1 gene in the Ag/Ca–Ti group was dramatically elevated when cocultured with macrophages. This result suggests that Ag/Ca–Ti samples did not have a significant osteogenic effect, whereas the osteogenic capacity of MC3T3-E1 was substantially enhanced after coculture with macrophages. This conclusion is also consistent with the findings in Fig. 5. The principle of the reaction associated with the cells on the surface of the material is shown in Fig. 8.

In summary, we primarily focus on the immunomodulatory effects of microcurrents, aiming to regulate immune responses and achieve immune-mediated osteogenesis through the Ag–Ca micro-galvanic system. This design strategy offers a novel approach for developing the next generation of anti-infective bone implants and demonstrates significant potential for future clinical translation. Furthermore, the above self-powered electrostimulation system holds great potential in clinical applications. On one hand, the in situ generated microcurrents can potentially be utilized for real-time clinical monitoring; on the other hand, ES can also be harnessed for therapeutic purposes, such as promoting wound healing and tissue regeneration.

## Conclusion

In this study, Ag–Ca micro-galvanic cells were fabricated on the surface of titanium (Ti) sheets using the plasma submerged immersion ion implantation technique to modulate cellular behavior through microcurrent effects. The effects of these materials on inflammation and osteogenesis were assessed through in vitro experiments. The Ag/Ca–Ti samples enhanced the proportion of M2-type macrophages and promoted the expression of anti-inflammatory genes. Furthermore, the Ag/Ca–Ti samples facilitated the expression of VGCC genes in the relevant cells via paracrine signaling from M2-type macrophages, leading to an increased influx of Ca^2+^ ions, which subsequently promoted osteogenesis and osteoblastic differentiation. Additionally, the Ag/Ca–Ti samples demonstrated excellent antibacterial properties, attributed to the microcurrents generated between Ag and Ca, as well as between Ag particles. This work provides valuable insights into the interplay between the microcurrent effects induced by multi-element doping and their impact on immune responses and osteogenic activity, offering new perspectives on the surface design of biomaterials.

## Materials and Methods

### Sample preparation

Medical titanium sheets of 1 mm thickness were cut into samples of 20 mm × 20 mm × 1 mm, 20 mm × 10 mm × 1 mm, and 10 mm × 10 mm × 1 mm. The 20 mm × 10 mm × 1 mm sample was used for the zeta potential test, while the remaining samples were 10 mm × 10 mm × 1 mm. The titanium samples were ultrasonically cleaned in a mixed acid solution (HF:HNO_3_:H_2_O = 1:5:34, v/v/v) twice, each time for 5 min, and the titanium samples were cleaned by ultrasonic cleaning with deionized water, ultrapure water, and ethanol. The sample, named Ti, was dried for use, and silver and calcium were used as cathodes for silver ion injection (Ag-PIII), calcium ion injection (Ca-PIII), and silver–calcium ion co-injection (Ag/Ca-PIII) on the surface of titanium samples by using a multifunctional ion implantation and deposition equipment. The injection parameters are shown in Table 1, and the samples with different injection times (hours) were named Ag–Ti, Ca–Ti, and Ag/Ca–Ti, respectively. During the injection process, the sample stage was cooled down using cooling circulating water and kept at 25 °C. The sample stage was then cooled down using the cooling circulating water.

**Table 1.** Equipment parameters for plasma immersion ion implantation

Parameters	Target (Ag)	Target (Ca)	Cathodic arc
Voltage pulse duration (μs)	800	1,000	500
Pulsing frequency (Hz)	10	10	10
Ion implantation voltage (kV)	−15	−15	
Ion implantation time (h)	1.5	1.5	
Pressure (Pa)	7.0 × 10^−2^		

### Characterizations

#### Surface structure and chemical composition

Scanning electron microscope (Hitachi S-4800) was used to analyze the morphology of the prepared samples, and the operating voltage was 15 kV and the current was 10 μA to test the surface micro-morphology of the materials. XPS was used to detect the chemical composition of the material surface.

#### X-ray photoelectron spectroscopy

XPS (K-Alpha+, Thermo Scientific) was used to detect the surface elements and the corresponding chemical states of each sample.

#### Hydrophilicity test

A video contact angle meter (Automatic Contact Angle Meter Model SL200B, Solon, China) was used to test the surface wettability of the samples. At room temperature, the samples were placed on the sample stage, and 2 μl of ultrapure water was added dropwise on the sample surface. After stabilization, photographs were taken, and the contact angle values were analyzed by the contact angle testing software. Three parallel samples were set in each group, and the mean ± SD was taken as the final result.

#### Zeta potential test

The zeta potential of the sample surface was measured using an electrokinetic solid surface analyzer (Surpass electrokinetic analyzer, Anton Parr, Austria). KCl (0.001 M) was used as the medium, and HCl (0.05 M) was used to adjust the pH.

#### Corrosion resistance

An electrochemical workstation (CHI760C, Shanghai Chenhua Instrument Co. Ltd.) was used to test the corrosion resistance of the samples. A 3-electrode electrochemical testing system was used: The sample to be tested was the working electrode, the saturated silver chloride electrode was the reference electrode, and the graphite electrode was the counter electrode. The electrolyte was 0.9% sodium chloride solution (pH 7.4, NaCl), the working voltage range was −0.5 to 0.5 V, the scanning rate was 0.01 V/s, the test area of the samples to be tested was 0.3 cm^2^, and the test was carried out after stabilization of the open circuit voltage. According to the polarization curves of the obtained samples, the corrosion potential and self-corrosion current were calculated to analyze the corrosion resistance of the sample surface.

#### Ion release

Samples measuring 10 mm × 10 mm × 1 mm were immersed in 10 ml of physiological saline at 37 °C for 1, 4, and 7 d without stirring. After each immersion, the solution was collected and stored at 4 °C and then replenished with fresh 10 ml of physiological saline. The number of released ions is determined by analyzing the resulting solution by inductively coupled plasma emission spectroscopy.

### In vitro studies

#### Immunological evaluation

##### Macrophage cell culture

Mouse monocyte–macrophage (RAW264.7, hereinafter referred to as macrophages) in vitro culture experiments were used to assess the effect of each group of samples on immune cell behavior. The cells were purchased from the Cell Resource Center of Shanghai Institutes for Biological Sciences, Chinese Academy of Sciences. The cell culture medium composition was 84% Dulbecco’s modified Eagle’s medium (DMEM) basal medium (high sugar, Gibco, USA), 15% fetal bovine serum (FBS; Gibco, USA), and 1% penicillin–streptomycin mixture (Gibco, USA). Macrophages were cultured in a constant temperature cell culture incubator (1L-161CT, Stuckey Instrumentation Co. Ltd.) at 37 °C with 5% CO_2_. The cells were passaged once every 2 to 4 d depending on the cell status. The samples used in the cell experiments were sterilized with 75% ethanol for 2 h in advance, the ethanol was replaced every 30 min, and the samples were dried on the ultra-clean table.

##### Cell proliferation

The proliferation of cells on the surface of the samples was tested using the Alma Blue kit. Sterilized samples were placed in 24-well cell culture plates (Thermo Fisher Scientific Inc., USA), and 1 ml of cell suspension with a density of 5 × 10^5^ cells/ml was added to each well; after incubation for 4 h, 1 d, and 4 d, the original culture medium was aspirated, the 24-well plate was replaced by a new one, and each well was washed twice with 0.6 ml of phosphate-buffered saline (PBS) for 5 min each time. The culture medium containing 10% alamarBlue infection solution was added and incubated for 2 h, 100 μl of each well was put into a black 96-well plate, and the fluorescence intensity of each well (excitation/emission wavelengths: 560/590 nm) was measured by using an enzyme labeling instrument (Synergy H4, Bio-Tek, USA). Four parallel samples were set for each group of samples, and the mean ± SD was taken as the cell proliferation results.

The morphology of cells on the sample surface was observed by SEM (S-3400N TypeI, Hitachi, Japan). After 4 h, 1 d, and 4 d of incubation, the culture medium was aspirated, and each well was washed 3 times with 0.6 ml of PBS, each time for 5 min; 0.6 ml of glutaraldehyde solution (2.5%, Sinopharm, Shanghai, China) was added, and the cells were fixed at 4 °C for 12 h, protected from light, and dehydrated with gradient ethanol (30, 50, 75, 90, 95, and 100 vol %). The samples were placed in a mixture of ethanol and hexamethylsilyl diaminane (HMDS; Sinopharm, Shanghai) in different volume ratios (volume of ethanol:volume of HMDS = 2:1, 1:1, 1:2, and 100% HMDS) in sequence and dried. SEM was used to observe the cell morphology.

##### Detection of immunity-related gene expression level (PCR experiment)

Real-time quantitative PCR (RT-qPCR) was used to detect the expression levels of immune-related genes in macrophages. Total RNA reagent was extracted using TRIzol (Invitrogen, Thermo Fisher Scientific Inc., USA). The expression levels of target genes relative to the internal reference were calculated according to the 2^−ΔΔCt^ relative quantitative analysis method using the housekeeping gene GAPDH (glyceraldehyde-3-phosphate dehydrogenase) as internal reference. Primers for RT-PCR are listed in Table S1 and were purchased from BioTNT.

##### Calcium pathway gene testing for RAW264.7

RAW264.7 cells were collected under both monoculture and coculture conditions. The expression levels of genes related to Ca^2+^ ion channels in macrophages were quantified using RT-qPCR. Total RNA was extracted using TRIzol reagent (Invitrogen, Thermo Fisher Scientific Inc.), with GAPDH serving as the internal control gene. Relative gene expression levels were calculated using the 2^−ΔΔCt^ method. The primers used for RT-qPCR are listed in Table S2 and were purchased from BioTNT.

#### Interactions between MC3T3-E1 and macrophages

##### Mouse embryonic osteoblast (MC3T3-E1) culture

In vitro culture experiments of mouse calvaria-derived pre-osteoblastic cell subclone E1 (MC3T3-E1) were used to evaluate the effect of each group of samples on the osteogenic differentiation of stem cells, and the cells were purchased from the Cell Resource Center of Shanghai Institutes for Biological Sciences, Chinese Academy of Sciences. The cell culture medium components were 85% DMEM basal medium (high sugar, Gibco, USA), 14% FBS (Gibco, USA), and 1% penicillin–streptomycin mixture (Gibco, USA). MC3T3-E1 was cultured in a constant temperature cell culture incubator (1L-161CT, Stuckey Instrumentation Co. Ltd.) at 37 °C with 5% CO_2_.

##### Coculture of MC3T3-E1 with macrophages

First, MC3T3-E1 was planted on the samples. After 3 d, RAW264.7 was then planted on the samples. On the fourth day, the samples planted with macrophages and the samples planted with MC3T3-E1 were cultured in the same environment, and the medium of both was mixed and added.

##### Detection of relevant gene expression levels (PCR experiment)

RT-qPCR was used to detect the expression levels of osteogenic genes associated with mouse calvaria-derived pre-osteoblastic cell subclone E1 (MC3T3-E1) employed. The operation was performed following the protocol described in “Detection of immunity-related gene expression level (PCR experiment)” section. Primers for RT-PCR are listed in Table S3 and were purchased from BioTNT.

##### Evaluation of osteogenic activity

ALP catalyzes the hydrolysis of phosphate bonds under alkaline conditions and is a by-product of osteoblasts. The samples were placed in 24-well culture plates, and 1 ml of cell suspension with a density of 1 × 10^4^ cells/ml was added to each well and incubated for 7 d. The samples were washed 3 times with PBS and placed in a new 24-well plate, and 0.5 ml of cell lysate was added to each well and lysed on ice for 40 min. The cell lysate was collected and centrifuged at 12,000 rpm for 15 min at 4 °C, and the resulting supernatant was discarded. The supernatant was centrifuged at 12,000 rpm for 15 min at 4 °C, and the reaction was terminated by adding p-nitrophenyl phosphate (p-NPP; Sigma, USA) and incubated at 37 °C for 30 min with 1 M NaOH solution. The absorbance at 405 nm was measured with an enzyme counter to calculate the total ALP activity. The total protein amount of the cells was detected using a constant bicinchoninic acid (BCA) kit (Thermo Fisher Scientific Inc., USA), and the ALP activity was expressed by the total ALP activity normalized by the total protein amount of the cells. Four samples were taken from each group, and the mean ± SD was taken as the final ALP activity result.

### In vivo studies

#### Animal experiment operation

Six-week-old male C57BL/6 mice were used, weighing about 20 to 30 g. Forty-eight mice were used in the formal experiments, randomly divided into 4 groups (Ti, Ag–Ti, Ca–Ti, and Ag/Ca–Ti) with 12 mice in each group. The mice were first pretreated, and the air sac inflammation model was constructed on the back of the mice. Upper back mouse hair was removed, and 4 ml of sterile air was injected subcutaneously to establish the air sac. On the fifth day, there was no obvious inflammatory reaction such as redness, swelling, exudate, pus, and hard nodule at the injection site, which indicated that the airbag was successfully constructed. On the seventh day, mice were anesthetized by intraperitoneal injection of 1% sodium pentobarbital. In a sterile environment, a surgical incision of about 1 cm in width was made at the upper edge of the air sac, and the samples were implanted into the air sac and sterilized with iodine povidone.

#### Histological analysis

The material-coated tissues were fixed in fixative for 24 h and then dehydrated and dipped in wax, followed by staining using H&E and Masson’s trichrome reagent, and then observed using a fluorescence microscope. CD206 and CD86 were selected as anti-inflammatory and anti-inflammatory markers, respectively, and the tissues were fluorescently stained and observed using a fluorescence microscope.

## Ethical Approval

All animal experiments were approved and carried out following the Institutional Animal Care and Use Committee. No human subjects were involved in this experiment.

## Data Availability

All data needed to evaluate the conclusion of this study are available on request.

## Supplementary Materials

Supplementary 1 Experimental Section Figs. S1 to S4 Tables S1 to S3
