# Supplementary material for: Construction of Silver–Calcium Micro-Galvanic Cell on Titanium for Immunoregulation Osteogenesis
Source: BME Front. 2025 Sep 8;6:0173. doi: 10.34133/bmef.0173 (PMC12415334; doi:10.34133/bmef.0173)
Supplement: Supplementary 1 — Experimental Section Figs. S1 to S4 Tables S1 to S3 [file bmef.0173.f1.docx]

**Supplementary Information**

**Construction of silver-calcium micro-galvanic cell on titanium for immunoregulation osteogenesis**

Zhenhao Hou^1,2,†^, Xingdan Liu^1,†^, Xianming Zhang^1^, Ji Tan^1,*^, and Xuanyong Liu^1,3*^

^1^ State Key Laboratory of High Performance Ceramics, Shanghai Institute of Ceramics, Chinese Academy of Sciences, Shanghai 200050, China

^2^ Center of Materials Science and Optoelectronics Engineering, University of Chinese Academy of Sciences, Beijing 100049, China

^3^ School of Chemistry and Materials Science, Hangzhou Institute for Advanced Study,

University of Chinese Academy of Sciences, Hangzhou, 310024, China

*Corresponding Author:

Dr. Xuanyong Liu, E-mail: xyliu@mail.sic.ac.cn

Dr. Ji Tan, E-mail: [tanji@mail.sic.ac.cn](mailto:tanji@mail.sic.ac.cn)

^†^The authors contributed equally to this work.

**Experimental Section**

*Macrophage Culture:* The effects of the samples on stem cell behavior were assessed using an in vitro culture of murine bone marrow-derived mesenchymal stem cells (mBMSCs). The mBMSCs were obtained from the Cell Resource Center, Shanghai Institute of Life Sciences, Chinese Academy of Sciences. The cells were maintained in a complete culture medium composed of 85% Mesenchymal Stem Cell Medium (MSCM) basal medium, 14% fetal bovine serum (FBS; Gibco, USA), and 1% penicillin-streptomycin solution (Gibco, USA). Macrophages were cultured at 37℃ with 5% CO₂ in a humidified incubator (1L-161CT, Siduke Instrument Equipment Co., Ltd., China). Cell passaging was performed every 2–4 days depending on cell growth status. Before cell experiments, all samples were sterilized with 75% ethanol for 2 hours, with ethanol refreshed every 30 minutes, and were air-dried in a biosafety cabinet.

*Cell Proliferation:* Cell proliferation on the sample surfaces was assessed using the alamarBlue™ assay. Sterilized samples were placed in a 24-well cell culture plate (Thermo Fisher Scientific Inc., USA), with each well seeded with 1 mL of cell suspension at a density of 1 × 10⁵ cells/mL. After 1, 4, and 7 days of culture, the medium was removed, and the samples were transferred to a new 24-well plate. Each well was washed twice with 0.6 mL phosphate-buffered saline (PBS) for 5 minutes per wash. Fresh medium containing 10% alamarBlue™ reagent was then added, and the plate was incubated in the dark for 2 hours. Subsequently, 100 μL of the supernatant from each well was transferred to a black 96-well plate, and fluorescence intensity was measured using a microplate reader (Synergy H4, Bio-Tek, USA) with excitation and emission wavelengths set at 560 nm and 590 nm, respectively. For each sample group, four parallel replicates were established, and the results were reported as mean ± standard deviation (SD).

*Direct Co-Culture of Macrophages and mBMSCs:* Initially, mBMSCs were seeded onto the surface of each sample at a density of 1 × 10^5^ cells per well. After 3 days of incubation, macrophages were seeded onto the same samples at a density of 1 × 10^6^ cells per well. Following an additional 24-hour incubation, the samples containing both macrophages and mBMSCs were combined into the same well, establishing a direct co-culture with shared culture medium. After 3 days of co-culture, the samples containing both cell types were carefully transferred to new plates for subsequent biological evaluations.

*Immunological Evaluation of Co-Cultured Macrophages:* The expression levels of immune-related genes in co-cultured macrophages were quantified using real-time quantitative polymerase chain reaction (RT-qPCR). Detailed procedures for this analysis are provided in Section 5.3.1.3.

*Osteogenic Evaluation of Co-Cultured mBMSCs:* The expression levels of osteogenesis-related genes in co-cultured mBMSCs were assessed using RT-qPCR. The detailed protocol for this assay is described in Section 5.3.2.3.

*In vitro Antibacterial evaluation:* Gram-negative Escherichia coli (ATCC 25922) and Gram-positive strains of Staphylococcus aureus (ATCC 25923) were used to evaluate the antibacterial activity of the samples. A 60 μL bacterial suspension at a concentration of 10^6^ CFU/mL was inoculated onto the surface of the sterilized samples (Ti, Ag-Ti, Ca-Ti, Ag/Ca-Ti) and incubated at 37 ℃ for 12 h. Subsequently, the bacterial solution was diluted with saline, and 100 μL of the diluted bacterial suspension was inoculated onto an agar plate and incubated at 37 ℃ for 18 h. To determine the survival rate of the bacteria, the bacterial suspension was incubated with saline for 10 min. In order to determine the survival rate of bacteria, the bacterial colonies were counted, and the formula for calculating the antibacterial rate was: antibacterial rate = (N_Control_-N_Experiment_)/N_Control_ × 100 %. In order to observe the morphology of the bacteria, the bacteria on the samples were fixed with 2.5% glutaraldehyde solution, dehydrated and dried, and the morphology of the bacteria was observed by SEM. 60 μL of the bacterial suspension at a concentration of 10^6^ CFU/mL was inoculated onto an agar plate, and the samples were placed on the agar plate and incubated at 37 ℃ for 18 h. Photographs of the agar plate were taken.


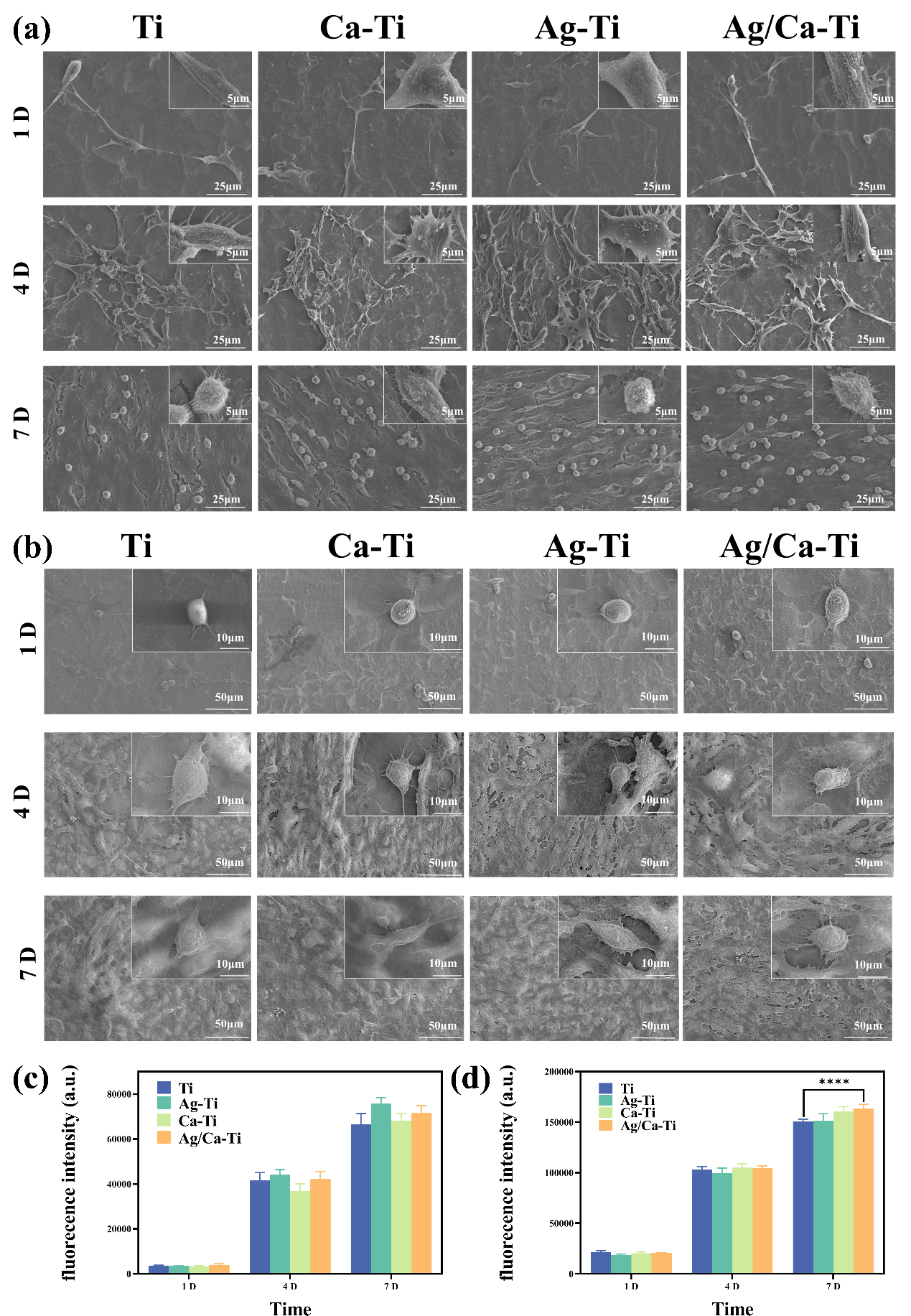


Fig. S1. Characterization of cell adhesion and proliferation of MC3T3-E1 and mBMSCs on different sample surfaces. Morphology of (a) MC3T3-E1 and (b) mBMSCs cultured on sample surfaces observed via SEM. Proliferation of (c) MC3T3-E1 and (d) mBMSCs on the surfaces.

Fig. S1(a) shows SEM images of MC3T3-E1 cells cultured on the surface of the samples, while Fig. S1(b) displays SEM images of mBMSCs. In all sample groups, both cell types exhibited good adhesion and spreading, with abundant lamellipodia and filopodia, indicating a favorable environment for cell proliferation. Remarkably, by day 7, the cells exhibited extensive spreading and formed a confluent monolayer on the sample surface, upon which a secondary layer of proliferating cells was observed, indicating sustained cellular activity and favorable biocompatibility. Fig. S1(c) and (d) present the proliferation data of MC3T3-E1 and mBMSCs on the sample surfaces. The proliferation data for the Ag/Ca-Ti group showed no significant reduction relative to the control and other experimental groups, suggesting that the Ag/Ca-Ti surface did not induce cytotoxic effects on either cell type.


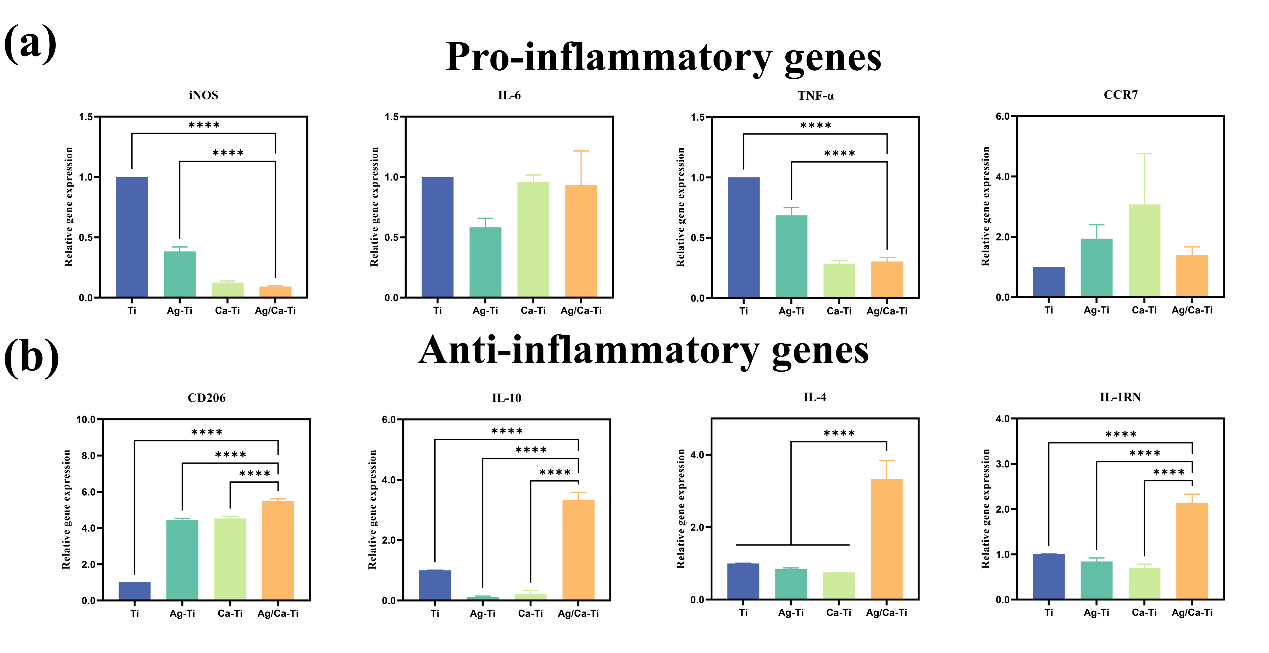


Fig. S2. Immunity-related gene expression results of RAW264.7 on the sample surface co-cultured with mBMSCs (a) pro-inflammatory genes, (b) anti-inflammatory genes.

Fig. S2 shows the experimental results of PCR technique to detect the expression levels of immune-related genes in sample macrophages co-cultured with mBMSCs. The results of the related genes showed that the immunoinflammatory tendency of RAW264.7 on Ag/Ca-Ti group samples would not be fundamentally changed with the participation of other cells, and it still showed a strong tendency to suppress inflammation. Moreover, compared with the culture alone, the co-culture state, the Ag/Ca-Ti samples promoted the inflammation-suppressing gene expression of RAW264.7 more obviously, and MRC1, IL-10, IL-4 and IL-1RN all showed gene expression effects far beyond those of other groups.


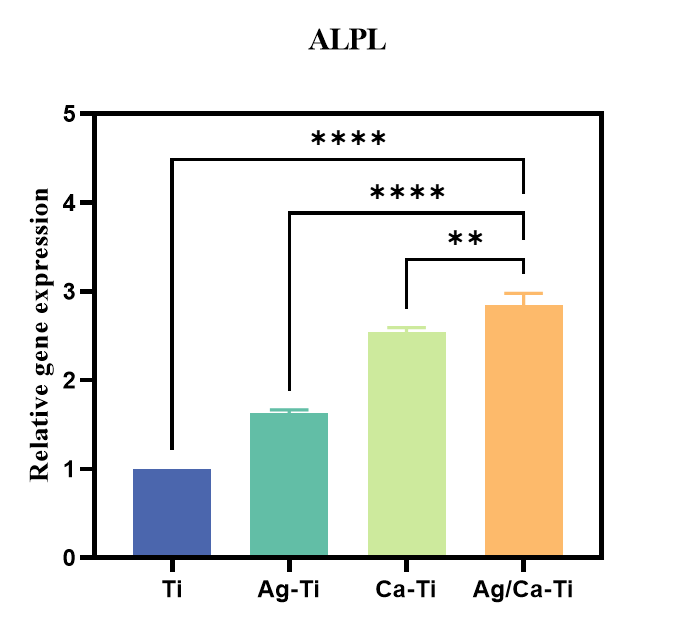


Fig. S3. mBMSCs expression of osteogenic gene in culture alone

Fig. S3 illustrates the expression levels of osteogenic genes in mBMSCs cultured alone. As shown in the figure, the expression of osteogenic-related gene in mBMSCs was upregulated on the Ag/Ca-Ti samples, indicating that Ag/Ca-Ti samples promote osteogenesis in mBMSCs.


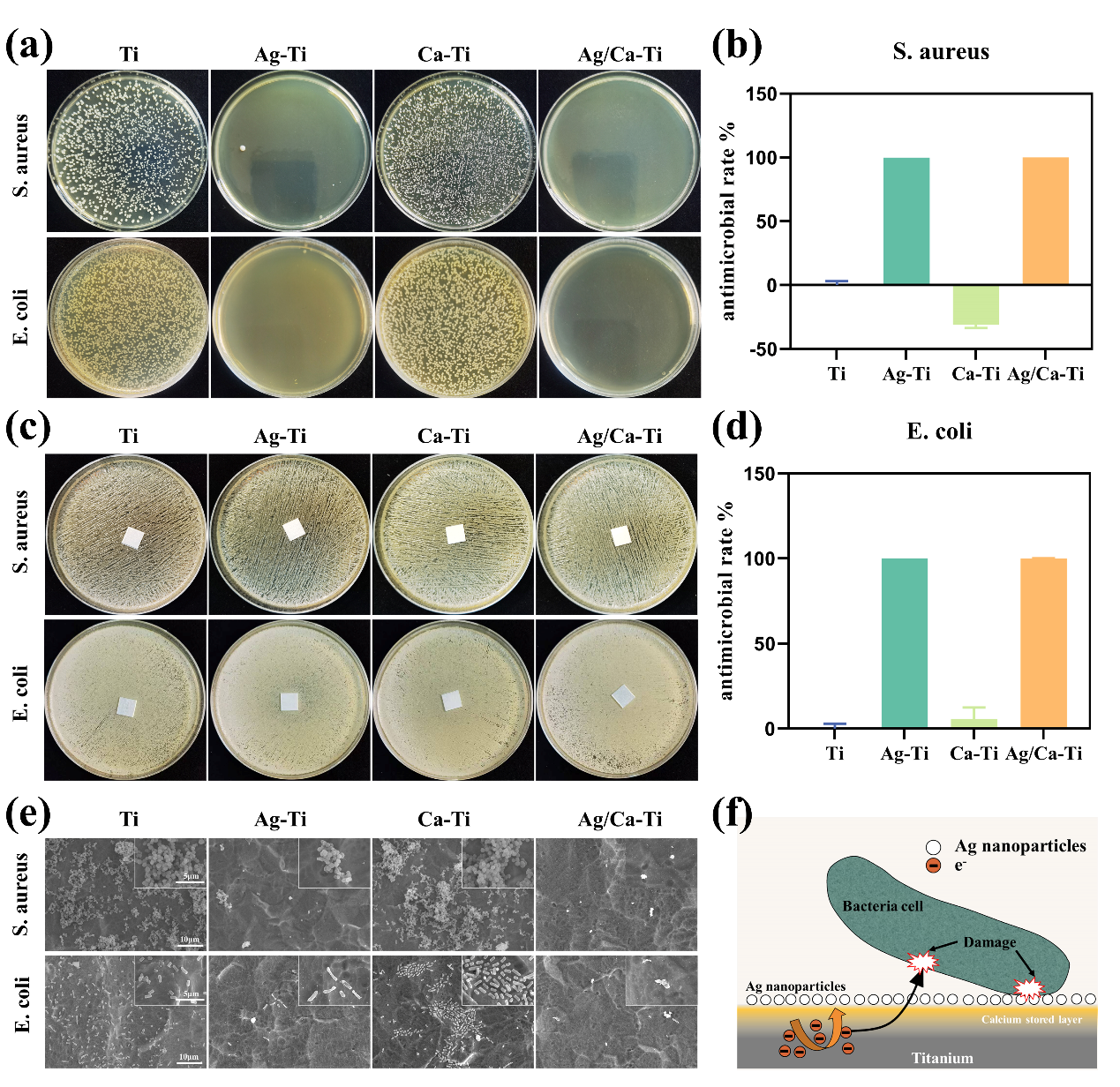


Fig. S4. Antibacterial effect of samples against bacteria: different samples against *Staphylococcus aureus* and *Escherichia coli*: (a) photographs of representative colonies of agar plates; (b) antibacterial rate of samples against *Staphylococcus aureus*; (c) photographs of representative rings of inhibition of agar plates; (d) antibacterial rate of samples against *Escherichia coli*; (e) morphology of *Staphylococcus aureus* and *Escherichia coli* on the surface of the samples; (f) antibacterial mechanism of the materials. antibacterial mechanism.

Fig. S4 shows the antibacterial properties of each group of samples against Staphylococcus aureus and Escherichia coli. From Fig. S4(a), it can be seen that Ti and Ca-Ti samples did not possess antibacterial properties while Ag-Ti and Ag/Ca-Ti samples possessed significant antibacterial properties. Fig. S4(b) and Fig. S4(d) also show that the activity of Ag-Ti and Ag/Ca-Ti samples against *Staphylococcus aureus* and *Escherichia coli* is close to 100%. The results of the ring of inhibition in Fig. S4(c) show that Ti, Ag-Ti, Ca-Ti and Ag/Ca-Ti showed no ring of inhibition in the Petri dishes of S. aureus, which indicates that the ion release from the surface of the material and the potential factors in the culture medium do not pose a threat to the survival of the bacteria. According to Fig. S4(e), it can be seen from the bacterial morphology on the surface of the materials as well as the number of *Staphylococcus aureus* and *Escherichia coli* that the number of bacteria on the surfaces of Ti and Ca-Ti samples is higher, and the number of bacteria on the surface of Ag/Ca-Ti is the least, and the bacterial structure is disrupted, which is also basically in agreement with the results of the bacterial coated plates.

**Table S1**. Primers used for real time-PCR in the immunological evaluation.

| Gene (mouse) | Primer sequences  (F, forward; R, reverse; 5’-3’) | Product size (bp) |
| --- | --- | --- |
| GAPDH | F: GCT CAG GCC TCT GCG CCC T  R: CCT ACT CTC TTG AAT ACC | 115 |
| CCR7 | F: ATC ATC CGT ACC TTG CTC CA  R: CAG GAC CAC CCC ATT GTA G | 117 |
| iNOS | F: TTG ACG CTC GGA ACT GTA  R: GTT GGT GGC ATA AAG TAT GTG | 74 |
| IL-1β | F: GAT ATT CTC CAT GAG CTT TG  R: ATT TAT TTA TGT ATT TAT T | 89 |
| IL-6 | F: CCA AGA GGT AAA AGA TTT AC  R: ATT GAT AAT TTA AAT AAG TA | 161 |
| TNF-α | F: TTA GAG CGG GAT AGT AAC G  R: CAA AAT ACA CAA CAG TGT C | 111 |
| CD206 | F: AGG GAA GAG AAG AAG ATC CAG  R: TGG GAG AAG ATG AAG TCA AAC | 107 |
| Arg-1 | F: GCC AGG GAC TGA CTA CCT TAA  R: AGT TCT GTC TGC TTT GCT GTG | 90 |
| IL-1rn | F: TGA ATC CTG TGA CCC TGT G  R: AAA CTG AAC CCC TGA GAA GAG | 97 |
| IL-4 | F: CCA TGA ATG AGT CCA AGT CC  R: TAA CTT ATG AAT TTT TAA T | 60 |
| IL-10 | F: CCC TTT GCT ATG GTG TCC T  R: GTG GCC AGT TTG TTA TTT AT | 106 |

**Table S2** Primers used for the RT-PCR in the Ca^2+^-related signaling pathway.

| Gene (mouse) | Primer sequences  (F, forward; R, reverse; 5’-3’) | Product size (bp) |
| --- | --- | --- |
| GAPDH | F: GCT CAG GCC TCT GCG CCC T  R: CCT ACT CTC TTG AAT ACC | 115 |
| Cav1.1 | F: CCA GCG TGT CTA TTC AGT TTC  R: CTC CTA AAT CAT CCC AGT CAG T | 112 |
| Cav1.2 | F: CTA GGC TTC GAG GAT CTG ATT  R: CTG ATT TCA AAG AGA GCA TGG | 89 |
| Cav1.3 | F: CAA AGG TCA GGT GAG CAT ACC  R: TTG AGG TGG AAT TGA GAG GAA | 87 |

**Table S3**. Primers used for real time-PCR in the osteogenesis evaluation.

| Gene (mouse) | Primer sequences  (F, forward; R, reverse; 5’-3’) | Product size (bp) |
| --- | --- | --- |
| GAPDH | F: GCT CAG GCC TCT GCG CCC T  R: CCT ACT CTC TTG AAT ACC | 115 |
| ALPL | F: GCA GGC AAG ACA CAG ACT  R: TGG AGG AGA GAA GGT CAG AT | 114 |
| RUNX-2 | F: GCA GCA CGC TAT TAA ATC CAA  R: GCC AAA CAG ACT CAT CCA TTC | 120 |
| TGF-β | F: AAG GAC CTG GGT TGG AAG T  R: GGT CCT TGC CCT CTA CAA C | 135 |
| RANKL | F: GGT TAA CCA AGA TGG CTT CTA  R: TAG TCT GTA GGT ACG CTT CCC | 84 |
